# Supplementary material for: Programming cell growth into different cluster shapes using diffusible signals
Source: PLoS Comput Biol. 2021 Nov 8;17(11):e1009576. doi: 10.1371/journal.pcbi.1009576 (PMC8601629; doi:10.1371/journal.pcbi.1009576)
Supplement: S1 Table — (PDF) [file pcbi.1009576.s001.pdf]

Summary of model parameters

Parameter	Definition/Description	Expression/Value in simulations
General		
μ_i	secretion rate of chemical i	$\mu_i(\vec{c}) = \mu_{i,max} \prod_j H_{ij}(c_j)$ (Eq 1)
$\mu_{i,max}$	maximum secretion rate of i,<br>can also depend on $\vec{c}$	N/A
H_{ij}	How chemical j affects the secretion rate of i	$H_{ij}(c_j) = \frac{c_j^{n_{ij}}}{K_{ij}^{n_{ij}} + c_j^{n_{ij}}}$
n_{ij}	Hill coefficient of H_{ij}	N/A
K_{ij}	concentration of j at which $H_{ij} = 0.5$,<br>can also depend on $\vec{c}$	 N/A
D_i	diffusion coefficient of chemical i	N/A
γ_i	degradation rate of chemical i	N/A
g	growth rate of cell	$g(\vec{c}) = \prod_j H_j(c_j)$ (Eq 3)
H_j	How chemical j affects the growth rate of the cell	$H_j(c_j) = \frac{c_j^{n_j}}{K_j^{n_j} + c_j^{n_j}}$
n_j	Hill coefficient of H_j	N/A
K_j	concentration of j at which $H_j = 0.5$<br>can also depend on $\vec{c}$	N/A<br>N/A
x_0	initial cluster length	N/A
y_0	initial cluster width	N/A
Example 1: Single growth inhibitor X (Fig 3)		
n_{XX}	How X affects its own secretion rate	0
μ_X	constant secretion rate of X	N/A
n_X	How X affects the growth rate of cells	-100
K_{gX}	Threshold concentration of X<br>above which cells stop growing	1
$\tilde{\mu}_X$	effective secretion rate $\frac{\mu_X}{\gamma_X K_{gX}}$	7, 8 or 9
$\tilde{x}_0$	scaled initial tissue length $\sqrt{\frac{\gamma_X}{D_X}} x_0$	1.5
$\tilde{y}_0$	scaled initial tissue width $\sqrt{\frac{\gamma_X}{D_X}} y_0$	1
Example 2: Two growth inhibitors X, Y (Fig 4)		
μ_X	constant secretion rate of X	N/A
μ_Y	secretion rate of Y	$\mu_Y(c_X) = \mu_{Y0} H_X(c_X)$ (Eq 6)
μ_{Y0}	maximum secretion rate of Y	N/A
n_{ij}	How i affects secretion rate of j	$n_{XY} = -100$<br>$n_{XX} = n_{YY} = n_{YX} = 0$
K_s	Threshold concentration of X<br>below which Y is secreted	0.9, 1, or 1.2

n_i	How $i = X, Y$ affects the growth rate of cells	$n_X = n_Y = -100$
K_{gi}	Threshold concentration of $i = X, Y$ above which cells stop growing	1
$\tilde{\mu}_X$	effective secretion rate of X: $\frac{\mu_X}{\gamma_X K_{gX}}$	8
$\tilde{\mu}_{Y0}$	effective maximum secretion rate of Y: $\frac{\mu_{Y0}}{\gamma_X K_{gY}} \frac{D_X}{D_Y}$	20-200
γ_r	rescaled degradation rate of Y: $\frac{\gamma_Y}{\gamma_X} \frac{D_X}{D_Y}$	1
$\tilde{x}_0$	scaled initial tissue length $\sqrt{\frac{\gamma_X}{D_X}} x_0$	1.5
$\tilde{y}_0$	scaled initial tissue width $\sqrt{\frac{\gamma_X}{D_X}} y_0$	1

Example 3: One growth inhibitors X with one growth-threshold regulator Y (Fig 5)		
μ_X	constant secretion rate of X	N/A
μ_Y	secretion rate of Y	$\mu_Y(c_X) = \mu_{Y,max}(c_X) H_X(c_X)$ (Eq 6)
$\mu_{Y,max}$	maximum secretion rate of Y	$\mu_{Y,max} = \mu_{Y0} \max(1 + bc_X, 0)$ (Eq 10)
b	how strongly X regulates $\mu_{Y,max}$	0, -0.5, -0.8
n_{ij}	How i affects secretion rate of j	$n_{XY} = 0, -100$<br>$n_{XX} = n_{YY} = n_{YX} = 0$
K_s	Threshold concentration of X below which Y is secreted	0.9, 1
n_i	How $i = X, Y$ affects the growth rate of cells	$n_X = -100; n_Y = 0$
K_{gX}	Threshold concentration of X above which cells stop growing	$K_{gX}(c_Y) = K_{gX0}(1 - ac_Y)$ (Eq 9)
K_{gX0}	baseline growth threshold of X (when Y is absent)	N/A
a	how strongly Y regulates K_{gX}	N/A
$\tilde{\mu}_X$	effective secretion rate of X: $\frac{\mu_X}{\gamma_X K_{gX0}}$	8
$\tilde{\mu}_{Y0}$	effective maximum secretion rate of Y: $\frac{a\mu_{Y0}}{\gamma_X} \frac{D_X}{D_Y}$	0.1-48
γ_r	rescaled degradation rate of Y: $\frac{\gamma_Y}{\gamma_X} \frac{D_X}{D_Y}$	0.2
$\tilde{x}_0$	scaled initial tissue length $\sqrt{\frac{\gamma_X}{D_X}} x_0$	1.5
$\tilde{y}_0$	scaled initial tissue width $\sqrt{\frac{\gamma_X}{D_X}} y_0$	1
