# Supplementary material for: Programming cell growth into different cluster shapes using diffusible signals
Source: PLoS Comput Biol. 2021 Nov 8;17(11):e1009576. doi: 10.1371/journal.pcbi.1009576 (PMC8601629; doi:10.1371/journal.pcbi.1009576)
Supplement: S1 Text — (PDF) [file pcbi.1009576.s002.pdf]

Solving for velocities at tissue boundary

In our simulations, the cells lie on a 2-dimensional regular grid. Given any tissue shape/outline, only grid points strictly inside the tissue contain cells that can grow and secrete chemicals. Solving the relevant reaction-diffusion equations (Eqs 1 and 2) on this 2-D grid gives the steady-state chemical concentrations. We then obtain the corresponding growth rates $g(\vec{x})$ (Eq 3) at each of these grid points.

The velocity of the tissue boundary given any spatial growth rate profile $g(\vec{x})$ is then found from solving the equation:

$$\nabla \cdot \mathbf{u} = g, \tag{Sa}$$

with $\mathbf{u} = -\nabla P$ and we impose the condition that the pressure is $P = 0$ at the cluster boundary.

To derive the boundary integral equation used for finding the boundary velocities, we follow closely the approach used in Ref. [1].

To deal with the boundary condition $P = 0$, we decompose the potential P into 2 components:

$$P = \hat{P} + \tilde{P}, \tag{Sb}$$

where

$$\hat{P}(z) = \int \frac{g(\tilde{z})}{2\pi} ln(|z - \tilde{z}|) d\tilde{z} \tag{Sc}$$

is the contribution from the growth rates of all cells within the tissue, while $\tilde{P}$ is the contribution due to the presence of the tissue boundary and satisfies:

$$\nabla^2 \tilde{P} = 0 \tag{Sd}$$

inside the tissue, with $\tilde{P} = -\hat{P}$ on the tissue boundary $\partial\Omega$.

The total cell velocity can therefore also be written as the sum of the two contributions: $\mathbf{u} = \hat{\mathbf{u}} + \tilde{\mathbf{u}}$, with $\hat{\mathbf{u}} = -\nabla \hat{P}$ and $\tilde{\mathbf{u}} = -\nabla \tilde{P}$. Since P is constant on $\partial\Omega$, the tangential component of the velocity $u_\tau = 0$, and to solve for the evolution of the tissue shape, we are interested in finding the normal velocity component $u_n = \tilde{u}_n + \hat{u}_n$.

To do so, it is convenient to consider $\partial\Omega$ as a contour of total length L in complex plane, and parameterise it with the arc length s such that that $z(s) = x(s) + iy(s)$ describes $\partial\Omega$ in the anticlockwise direction with $0 \leq s \leq L$.

The unit normal and tangent vectors on $\partial\Omega$ are then given by:

$$\vec{n} = \left(\frac{dy}{ds}, -\frac{dx}{ds} \right) \tag{Se}$$

$$\vec{\tau} = \left(\frac{dx}{ds}, \frac{dy}{ds} \right). \tag{Sf}$$

To reformulate Eq Sd in terms of a complex contour integral, we define the streamline function $\tilde{\psi}$ such that $\frac{\partial \tilde{\psi}}{\partial y} = \frac{\partial \tilde{P}}{\partial x}$ and $\frac{\partial \tilde{\psi}}{\partial x} = -\frac{\partial \tilde{P}}{\partial y}$ (and hence $\nabla^2 \tilde{\psi} = 0$). The complex potential $f(z) = \tilde{P}(z) + i\tilde{\psi}(z)$ is then an analytic function, and so is its derivative $\frac{\partial f}{\partial z} = w = \tilde{u} - i\tilde{v}$, where $\tilde{u}$ and $\tilde{v}$ are respectively the x and y components of $\tilde{\mathbf{u}}$.

We then define the function $F(z) = \frac{\partial f / \partial z}{z - z_m}$, such that $F(z)$ is a analytic function inside the tissue except at the simple pole $z = z_m$. If the pole z_m lies on the boundary, based on Cauchy's integral formula,

$$\oint_{\partial\Omega} F(z) dz = i\pi \left. \frac{\partial f}{\partial z} \right|_{z_m}, \tag{Sg}$$

which can be rewritten as:

$$\int_0^L \frac{\tilde{u}(s) - i\tilde{v}(s)}{z(s) - z(s_m)} \frac{dz}{ds} ds = i\pi (\tilde{u} - i\tilde{v}) \Big|_{s_m}. \tag{Sh}$$

Since $(\tilde{u} - i\tilde{v}) \frac{dz}{ds} = \tilde{u}_\tau + i\tilde{u}_n$, this can be expressed as:

$$\int_0^L \frac{\tilde{u}_\tau(s) + i\tilde{u}_n(s)}{z(s) - z(s_m)} ds = i\pi (\tilde{u}_\tau(s) + i\tilde{u}_n(s)) \left(\frac{dz}{ds} \right)^{-1} \Bigg|_{s_m}. \tag{Si}$$

since $P = 0$ on $\partial\Omega$, $\tilde{u}_\tau = -\hat{u}_\tau$ on the tissue boundary. We therefore have

$$\fint_0^L \frac{-\hat{u}_\tau(s) + i\tilde{u}_n(s)}{z(s) - z(s_m)} ds = i\pi(-\hat{u}_\tau + i\tilde{u}_n) \left(\frac{dz}{ds}\right)^{-1} \Bigg|_{s_m} \tag{Sj}$$

for any $0 \leq s_m \leq L$, and $\hat{u}_\tau(s)$ known from Eq Sc.

By discretizing the boundary and setting s_m to be the mid-points of the mesh, these set of equations can be solved numerically to obtain $\tilde{u}_n$ at each of the discrete mesh points. The total velocity is then found by adding this to $\hat{u}_n$ (which is also known from Eq Sc).

[1] A. Khalid, N. McDonald, and J.-M. Vanden-Broeck, “On the motion of unsteady translating bubbles in an unbounded hele-shaw cell,” *Physics of Fluids*, vol. 27, no. 1, p. 012102, 2015.
