# Supplementary material for: Programming cell growth into different cluster shapes using diffusible signals
Source: PLoS Comput Biol. 2021 Nov 8;17(11):e1009576. doi: 10.1371/journal.pcbi.1009576 (PMC8601629; doi:10.1371/journal.pcbi.1009576)
Supplement: S2 Text — (PDF) [file pcbi.1009576.s003.pdf]

Example of a regulatory mechanism involving a growth activator

We consider here an example where all cells secrete a chemical Y at a constant rate μ_Y, and Y inhibits the secretion of a growth activator X (panel a in S2 Fig). This implies that X is only secreted when the concentration of Y is below a threshold K_s, and cells only divide when the concentration of X is above a threshold K_{gX} (panel a in S2 Fig).

The steady-state concentrations of X and Y satisfy the following set of non-dimensional equations:

$$\tilde{\nabla}^2 \tilde{c}_X + \tilde{\mu}_X(\tilde{c}_Y) - \tilde{c}_X = 0$$
$$\tilde{\nabla}^2 \tilde{c}_Y + \tilde{\mu}_Y - \gamma_r \tilde{c}_Y = 0$$

where the rescaled length scale $\tilde{x} = \sqrt{\frac{\gamma_X}{D_X}} x$, rescaled concentrations $\tilde{c}_X = \frac{c_X}{K_{gX}}$, $\tilde{c}_Y = \frac{c_Y}{K_s}$, effective secretion rates $\tilde{\mu}_X = \tilde{\mu}_{X0} H_{XY}(\tilde{c}_Y|1, -100)$ with $\tilde{\mu}_{X0} = \frac{\mu_X}{\gamma_X K_{gX}}$ and $\tilde{\mu}_{Y0} = \frac{\mu_{Y0}}{\gamma_X K_s} \frac{D_X}{D_Y}$, and the rescaled Y degradation rate $\gamma_r = \frac{\gamma_Y}{\gamma_X} \frac{D_X}{D_Y}$.

With a given parabolic initial cell cluster, if $\tilde{\mu}_{Y0}$ is too high, none of the cells will be producing X and hence no cells will be dividing. For intermediate values of $\tilde{\mu}_{Y0}$, only cells near the tip can produce X, and hence any growth zone will be localized at the tip. This can give rise to a protrusion (panel b in S2 Fig).
