# Supplementary figures and images for: Programming cell growth into different cluster shapes using diffusible signals
Source: PLoS Comput Biol. 2021 Nov 8;17(11):e1009576. doi: 10.1371/journal.pcbi.1009576 (PMC8601629; doi:10.1371/journal.pcbi.1009576)

(a)

$X$

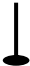

*growth*

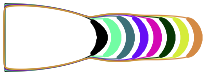

(b)

$X$

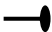

$Y$

*growth*

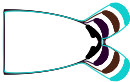

(c)

$X$

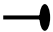

$Y$

*growth*

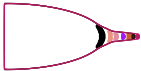

Supplement: S1 Fig — (a) Single growth inhibitor [parameters: μX˜=7], (b) 2 growth inhibitors [parameters:μ˜X=7, μ˜Y0=59, K˜s=1, γr = 1], and (c) 1 growth inhibitor and 1 growth-threshold regulator [parameters:μ˜X=7, μ˜Y=50, K˜s=0.9, γr = 0.2, b˜=-0.8]. [Other parameters: initial tissue length x˜0=1.5, initial tissue width y˜0=1]. (PDF) [file pcbi.1009576.s004.pdf]

(a)

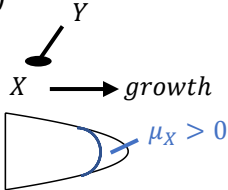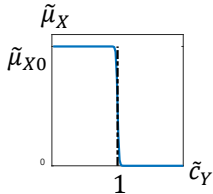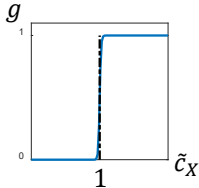

(b)

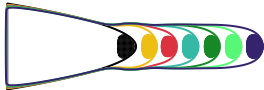

Supplement: S2 Fig — (a) All cells secrete Y, which inhibits the secretion of a growth activator X. (b) Example of growth dynamics with this regulatory scheme giving rise to a protrusion. [parameters:μ˜X0=48, μ˜Y=7/1.2, γr = 0.5, initial tissue length x˜0=1.5, initial tissue width y˜0=1 (see S2 Text for details of model description)]. (PDF) [file pcbi.1009576.s005.pdf]

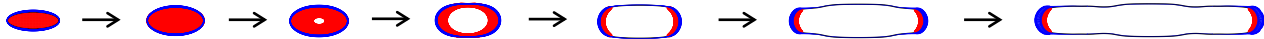

Supplement: S3 Fig — Starting with a small elliptical cluster, initially none of the cells are inhibited (red regions represent growth zones) and the cluster expands in all directions (boundary velocities indicated by the thickness of the blue region perpendicular to the tissue surface). As the number of cells increases, some of the cells stop dividing as concentration of the growth inhibitor exceeds a threshold. The tissue eventually elongates only along the horizontal axis as the growth zone becomes restricted to the tips of the cluster. [Parameters: μeff = 7, initial tissue length x˜0=1.6, initial tissue width y˜0=0.4.]. (PDF) [file pcbi.1009576.s006.pdf]

(a)  $\widetilde{K}_s = 0.9,$   
 $\widetilde{\mu}_{Y0} = 150$

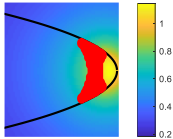

(b)  $\widetilde{K}_s = 1,$   
 $\widetilde{\mu}_{Y0} = 56$

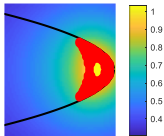

(c)  $\widetilde{K}_s = 1.2,$   
 $\widetilde{\mu}_{Y0} = 20$

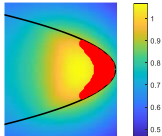

Supplement: S4 Fig — With the 2 growth inhibitors regulatory scheme (in Fig 4a), for a fixed μ˜X=8, the shape of the growth zone depends on the concentration profile of growth inhibitor Y. The background colors (with the corresponding legends) represent c˜Y. Growth is inhibited when c˜Y>1, as can be seen from the shapes of the growth zones (indicated by the regions in red). [Other parameters: γr = 1, initial tissue length x˜0=1.5, initial tissue width y˜0=1.]. (PDF) [file pcbi.1009576.s007.pdf]

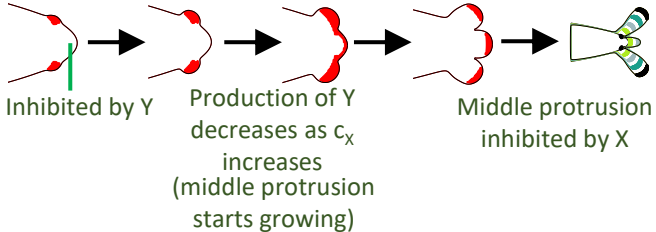

Supplement: S5 Fig — [Parameters: μ˜X=8, μ˜Y=65, K˜s=1 γr = 1, initial tissue length x˜0=1.5, initial tissue width y˜0=1.]. (PDF) [file pcbi.1009576.s008.pdf]

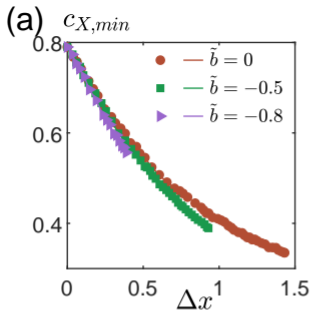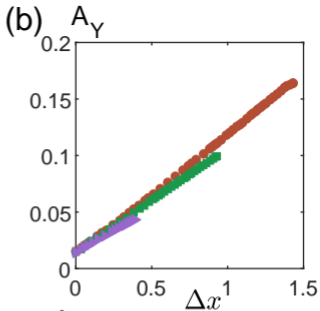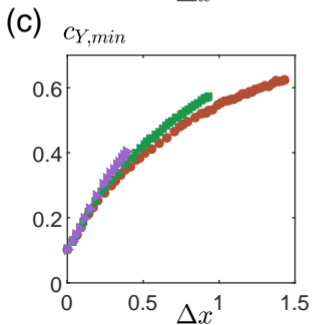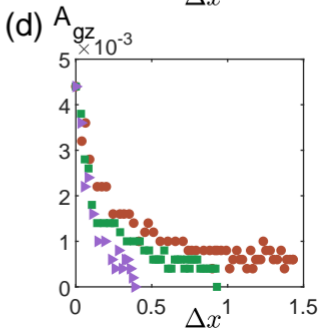

Supplement: S6 Fig — (a) As the tissue elongates, the minimum concentration cX,min of X at the tissue tip decreases. (b) Since X inhibits the secretion of Y, a reduction in cX increases the area AY within which cells are secreting Y. (c) As AY increases, cY at the tissue tip also increases. Since a higher |b˜| implies a larger increase in the secretion rate μ˜Y,max of Y as cX decreases, cY increases faster when |b˜| is larger. (d) Since Y reduces the growth threshold of X, an increase in cY reduces the area Agz of the growth zone, with a faster decrease when |b˜| is larger. [Other parameters: μ˜X=8, μ˜Y={15,whenb˜=026.5,whenb˜=-0.548,whenb˜=-0.8, K˜s=0.9 γr = 0.2, initial tissue length x˜0=1.5, initial tissue width y˜0=1.]. (PDF) [file pcbi.1009576.s009.pdf]

(a)

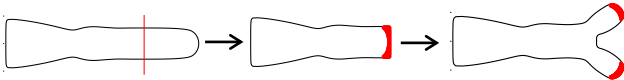

(b)

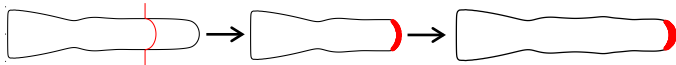

Supplement: S7 Fig — (a) With a straight cut (red vertical line) perpendicular to the protrusion (left), the cluster grows two protrusions (right). This arises because compared to the original rounded edge of the protrusion, there are more cells at the corner and they are uninhibited by the growth inhibitor (middle). Red region indicates the growth zone. (b) WIth a curved (elliptical) cut (left), a single protusion reemerges (right) from a growth zone at the tip of the protrusion (middle). [Parameters: μ˜X=8.]. (PDF) [file pcbi.1009576.s010.pdf]
