# Supplementary material for: Programming cell growth into different cluster shapes using diffusible signals
Source: PLoS Comput Biol. 2021 Nov 8;17(11):e1009576. doi: 10.1371/journal.pcbi.1009576 (PMC8601629; doi:10.1371/journal.pcbi.1009576)
Supplement: Attachment — Submitted filename: response_to_reviewers.pdf [file pcbi.1009576.s011.pdf]

Dear Editor,

Thank you for considering our work for *PLOS Computational Biology* and for handling our manuscript.

We thank the reviewers for their positive evaluation and their constructive comments and suggestions for our manuscript, “Programming cell growth into different cluster shapes using diffusible signals”, which we have revised accordingly. We have addressed all comments raised by the reviewers, and have made the required revisions in the manuscript and supplementary material. Please find our point-by-point response below. We look forward to hearing from you.

Yours sincerely,

Yipei, Mor, and Michael.

Reviewer #1: The manuscript by Guo et al. presents a modeling framework for the analysis of tissue growth patterning in a concentration vector field of several interacting chemical species regulating tissue growth. Essentially, the approach treats the tissue as a continuous field, with local differences in growth rate that is determined by superposition of all growth factors, which are described by a standard reaction-diffusion model. The authors then restrict themselves to the analysis of models with one or several growth inhibitors, and find that growth inhibitors can induce growth of protrusions, the number of protrusions scaling exponentially with the number of growth inhibitors.

The authors present an innovative modeling framework, and therefore the paper seems appropriate for the journal. However, the analysis and results sections fall somewhat short, especially in light of the broad questions and claims suggested in the abstract and introduction. For proof of principle of the modeling framework, a minimal requirement would be an example with a growth stimulator, and a straight-forward case would also be a combination of a growth inhibitor and a growth stimulator with varying degree of interaction. Furthermore, it would be appropriate to spell out at least one of the examples mentioned in the introduction (notch receptor, Hedgehog secretion, synthetic activator-inhibitor system) in terms of a more specific model.

We thank the reviewer for the careful reading of the manuscript and the useful comments.

We include a section to show that within our model, if all cells divide at the same rate (with no growth regulation), an elongated initial cluster will become increasingly circular as it grows (section B and Fig. 2 in the revised manuscript). This is analogous to the case where cells only secrete a growth activator, since as the number of cells increases, the activator concentration increases, and therefore the number of growing cells also increases. The shape of the tissue will therefore become increasingly circular in this example. Hence, we focus on regulatory mechanisms where growth regulators are inhibitors rather than activators. This is discussed in lines 275-287 in the revised manuscript. Nevertheless, we now show in the SI an example of how

a regulatory mechanism involving a growth activator can be used to grow a protrusion if cells secrete another chemical that inhibits the secretion of this growth activator (SI section IV, Fig. S2), and refer to this in the main text lines 288-292.

The examples listed in the second paragraph of the introduction are of engineered pattern formation in a static group of cells, rather than the regulation of growth that generates different tissue shapes. There are natural systems in which growth rate is regulated by diffusible chemicals. For example, in growing bird beaks, there is a region enriched with BMP growth factors near the tip of the beak known as the growth zone in which cells are actively proliferating. In plant leaves, the chemical auxin activates growth while the chemical CUC2 is a growth repressor. The concentration profiles of these chemicals therefore determine the spatial profile of growth rate across the leaf, and hence the resulting leaf shape. We now include these examples in the introduction (lines 101-109), which serve as motivation for our study.

Further comments:
p.2, “we model the cell cluster as an incompressible cellular fuid of constant density…” – really? From my understanding, Eq 4 is the opposite of an incompressible fluid which is defined by vanishing divergence. Would Eq. 4 not rather suggest an analogy to an electric field where g would correspond to the charge density?

In our model, the cell cluster is incompressible since the density of cells within the cluster is assumed to be constant. Any non-zero divergence of the velocity arises only because of the division of cells. This is now emphasized in lines 188-190. Indeed, one can also think of the fluid containing a field of growth sources (which are the growing cells, analogous to positive charges in an electric field).

p. 3, “Inspired by the example of developing bird beaks … we choose to initialize the system with a 2-dimensional parabolic cell cluster” – not clear why bird beaks should be the reference point here. Could the authors discuss the choice of the region and initial conditions in light of the examples given in the introduction? Furthermore, a model scheme illustrating the simulated region and starting conditions would be very helpful.

We now describe in the introduction developing bird beaks as an example of how diffusible morphogens regulate a shape of a tissue (lines 102-105). This contrasts with the other examples of Hedgehog-secreting cells, synthetic activator-inhibitor circuits etc., which are examples of how pattern-formation can occur within a fixed array of cells rather than how the shape of the overall tissue can be regulated.

We now also explicitly state that in the spirit of developing bird beaks, a parabolic initial cell cluster is chosen because we are interested in exploring the growth of protrusions from the tip of the cluster (lines 234-239). We also include a panel in Fig. 1 to illustrate our choice of the initial tissue shape (Fig. 1b).

Reviewer #2: Guo et al, have made use of simulation studies in order to provide insights into the complexity of signals required and how they can be programmed to enable shape formation by tissues. They look into the case of morphogen gradients and growth-based shape formation. Their simulation studies indicate that it is possible to form a rod-shaped structure using just one growth inhibitory signal. In addition, they show that using more than one growth inhibitory signals, it is possible to allow the formation of several protrusions. Finally, they illustrated that the shape and length of each protrusion can be influenced by threshold regulators. This is an important topic and the paper and approach taken are of interest for the emerging field of multicellular systems engineering.

We thank the reviewer for the positive assessment of the manuscript.

Please find my comments below:
1) While the model has attempted to explain process of elongation (initiation of protrusion, numbers and also forms), there is less information about possible ways to initiate and terminate the formation of protrusion. This can be discussed, and possible models can be provided in 3 sets:
a) How to terminate formation of morphogenesis ?
b) How to initiate the process? how the inhibitory access is established in short range
c) How the process is maintained (and moves forward)? (perhaps this is the part that is already covered)

We thank the referee for this helpful comment and framing.

With a single growth inhibitor, we find that the single protrusion can grow indefinitely. In this case, other mechanisms are therefore required to terminate the growth of the multicellular cluster. This can occur, for example, when cells encounter depletion in nutrients or other external signals necessary for growth. This is discussed in lines 346-351. We also explore the growth of cone-shaped protrusion as an example of autonomous termination of protrusion growth (lines 359-363, lines 479-480).

With regards to the initiation of protrusions, we have assumed that there exists some initial form of symmetry-breaking mechanism (e.g., external concentration gradient) that gives rise to an asymmetric initial cell cluster (lines 218-222). If all cells in the initial cluster are dividing (i.e., concentration of growth inhibitor is low such that none of the cells are growth-inhibited), the cluster will initially grow in all directions. When the cluster becomes large enough, the growth zone can become localized at the tip of the tissue which would lead to a formation of an elongated protrusion. We discuss this in lines 319-327 and illustrate this in Fig. S3.

2) Would the model support “regeneration” of protrusion after injury? In discussion please also add how the model may explain how/why regeneration can fail in some cases of tissue injury or in some species.

Within our model, if one were to cut part of the protrusion, the subsequent dynamics would depend on the cut surface. For example, when cells secrete a single growth inhibitor, a straight cut perpendicular to the elongation axis of the protrusion would give rise to two protrusions due to non-growth-inhibited cells at the corners of the cut, while a curved cut that protrudes in the middle would lead to regeneration of a single protrusion. In the revised manuscript, we illustrate this in the new Fig. S7. This is consistent with the fact that during the growth of a single protrusion, the tip of the protrusion remains curved throughout growth. What types of regulatory circuits promote regeneration and what conditions are required for regeneration is an interesting question we leave for future work. This is now discussed in the lines 688-703.

3) Line 1 to 3: Synthetic biology is a broad term, and it would be more accurate to say: A fundamental goal of synthetic developmental biology is to understand how cells can be programmed to generate a desired spatial configuration.

Thank you for this comment. This is now corrected (line 44).

4) For easier read, the authors are expected to clearly annotate all the parameters used in their equations in a consistent manner and provide a clear word description of each of the parameters used (perhaps in a separate table).

Thank you for this comment. We now include a table in the SI with a list of parameters and their descriptions (SI section I, Table S1), and refer to it in the text in lines 176-177.

5) Authors mention :" we illustrate how our approach can be used to guide the design of regulatory circuits for achieving a desired target structure". Please elaborate in abstract and in text about this notion. It is not clear if they really achieve this. Perhaps either tune down the claim or provide more explanation for readers to truly prove ability to guide the design. Unfortunately since there is no wet lab experiment to show accuracy of prediction this calim should be done carefully.

We tune down this claim and elaborate on this point in the abstract by saying that "We show that a single growth inhibitor is insufficient for the formation of multiple protrusions but may be achieved with multiple growth inhibitors, and that other types of signals can regulate the shape of protrusion tips. These examples illustrate how our approach could potentially be used to guide the design of regulatory circuits for achieving a desired target structure." (lines 15-19) We also state in the Discussion Section (lines 673-675) that our model provides predictions that could potentially be tested in the lab in the future.

6) The authors are expected to compare and contrast their proposed models with other previously established models in the discussion section. What are the strength and weaknesses that each model has and what part of in vivo processes are not met with current models. Additionally, for example: they build up their model based on the tip proliferating cells (distal cells) that grow and become farther from the source inhibitory signal. Would it be possible to think about proximal cells

(at base) to grow and promote elongation and an activator signal that is produced by more distal cells.

In this paper, we adopt a framework which couples possible growth regulatory mechanisms to the overall expansion of the tissue. Given any regulatory circuit, the spatial chemical concentration profiles are modelled using the standard reaction-diffusion equations, and the corresponding growth rate of individual cells are specified as functions of these concentrations. The growth of the overall cell cluster is then obtained by treating it as an incompressible fluid, an approach that has been previously proposed and used to model a wide variety of systems ranging from the development of multicellular tissues [refs 29-35] to the expansion of bacterial colonies [refs 36-38]. The purpose of many of these studies is to recapitulate and hence understand a specific observation in a system. This requires incorporating details specific to that system-of-interest. For example, models of chick limb development involve specifying distinct regions (i.e., containing different cell types) within the tissue, namely the zone of polarizing activity and the apical epidermal ridge, that are separately responsible for producing the diffusible proteins Sonic Hedgehog (Shh) and fibroblast growth factors (FGFs) respectively [refs 29-30]. The concentrations of Shh and FGFs affect both the secretion rates of each other, and the proliferation rate of cells [refs 29-30]. In contrast, our goal here is to use this simple class of models to explore what is possible and how complex the regulatory schemes need to be when there is just a single cell type whose growth rate is regulated by diffusible morphogens alone. This is discussed in lines 598-626.

Within our model, we assume that all cells are of the same type, and cells exhibit different phenotypes (i.e., whether they grow or secrete a certain morphogen) based only on differences in their local environments. A potential mechanism for a growth activator A to be secreted only by (distal) cells at the tip is one where all cells are secreting chemical B which inhibits the secretion of A (since concentration of B will be higher away from the tip). If A is secreted only by the distal cells, it will form a growth zone localized near the tip which can generate a protrusion. In the revised manuscript we illustrate this in the new SI section IV, Fig. S2, and discuss this in lines 288-292.
