# Supplementary material for: Programming cell growth into different cluster shapes using diffusible signals
Source: PLoS Comput Biol. 2021 Nov 8;17(11):e1009576. doi: 10.1371/journal.pcbi.1009576 (PMC8601629; doi:10.1371/journal.pcbi.1009576)
Supplement: Attachment — Submitted filename: response_to_reviewers.pdf [file pcbi.1009576.s012.pdf]

Dear Editor,

Thank you for your attention to our manuscript. We are delighted to learn that our changes satisfied the reviewer.

We modified the manuscript to address the additional comment mentioned in the re-review (editing two paragraphs in the Introduction; see below). Please find our response to the reviewer below.

We look forward to hearing from you.

Yours sincerely,

Yipei, Mor, and Michael

Reviewer #1: The paper improved substantially in revision, and the authors have addressed most of my points. Especially, adding the case without growth regulation and adding more references to previous work was very helpful. However, I am still somewhat confused about the scope of the paper, and I would ask the authors to clarify that before publication.

In particular, the authors wrote in response to my first review:
“We now describe in the introduction developing bird beaks as an example of how diffusible morphogens regulate a shape of a tissue. This contrasts with the other examples of Hedgehog-secreting cells, (…)”.

On the other hand, in the revised introduction, there is no clear distinction between the two types of examples, nor are any conceptual differences pointed out. Rather, the introduction still gives the impression that all mentioned examples can be treated with the theoretical concept presented in the manuscript. I now understand that this is not the case, which should be clearly stated and discussed.

Furthermore, I would expect already in the introduction a reference to previous work using similar concepts (such as lines 605-609 in the discussion), as well as a statement regarding the “goal” (line 622), the novelty and the scope of the presented approach.

We thank the reviewer for these suggestions.

We now clarify the difference between the two types of examples by saying that “while spatial patterning only involves the spatial arrangement of cells with different gene expression profiles, achieving a desired tissue morphology requires that differences in gene expression lead to downstream physiological effects such as changes in the growth/proliferation or death rates of cells.” (lines 78-83)

We include, in the revised introduction, a description of how we model the growth of the cell cluster by treating the tissue as an incompressible fluid, together with references to previous work using similar concepts (lines 137-141). We also state that within our simple class of models, “we explore what is possible and how complex the regulatory schemes need to be when there is just a single cell type whose growth rate is regulated by diffusible morphogens alone.” (lines 141-144)
